# A New Species of Frog (Anura: Dicroglossidae) Discovered from the Mega City of Dhaka

**DOI:** 10.1371/journal.pone.0149597

**Published:** 2016-03-02

**Authors:** Mohammad Sajid Ali Howlader, Abhilash Nair, Juha Merilä

**Affiliations:** Ecological Genetics Research Unit, Department of Biosciences, University of Helsinki, Helsinki, Finland; Universita degli Studi di Roma La Sapienza, ITALY

## Abstract

We describe a new species of frog of the genus *Zakerana* discovered from the urban core of Dhaka, Bangladesh, one of the most densely populated cities in the world. Although the new species is morphologically similar to the geographically proximate congeners in the Bangladeshi cricket frog group, we show that it can be distinguished from all congeners on the basis of morphological characters, advertisement calls and variation in two mitochondrial DNA genes (12S rRNA and 16S rRNA). Apart from several diagnostic differences in body proportions, the new species differs from other *Zakerana* species in having a flattened snout (from ventral view) projecting over the lower jaw, and diagnostic trapezoid-shaped red markings on the vocal sac in males. Molecular genetic analyses show that the new species is highly divergent (3.1–20.1% sequence divergence) from all congeneric species, and forms a well-supported clade with its sister species, *Zakerana asmati*. The discovery of a new amphibian species from the urban core of Dhaka together with several recent descriptions of new amphibian species from Bangladesh may indicate that more amphibian species remain to be discovered from this country.

## Introduction

The rapid expansion of human activities, including urbanization, is widely recognized as a threat to local biodiversity (e.g., [1–4]). Habitat loss and fragmentation have reduced population sizes and led to extinctions of many amphibian species in anthropogenically altered landscapes (e.g., [5–9]). In fact, modern agricultural and forest management threatens over one-third of the world’s known amphibian species [10], and amphibian populations are facing severe persistence problems especially in mega cities [11–15].

The amphibian genus *Zakerana* is comprised of 20 recognized species [16,17]. After reviewing all previous studies and available morphological data (e.g., [18–33]), Howlader [34] reported five geographically proximate species of *Zakerana* from Bangladesh (*viz*. *Z*. *nepalensis*, *Z*. *pierrei*, *Z*. *syhadrensis*, *Z*. *teraiensis* [also distributed in India and Nepal] and *Z*. *asmati*). In the present paper we describe a new species of *Zakerana* from the urban core of Dhaka (the capital of Bangladesh), one of the most densely populated mega cities in the world [35]. While the new species is morphologically similar to the above-mentioned five congeners, we show that it can be distinguished on the basis of significant differences in morphology, bioacoustics, and genetic variability in two mitochondrial genes (16S and 12S rRNA). Given that Dhaka continues to grow and develop seemingly without any particular attention to the loss and degradation of its wetland ecosystems [36,37], we hope that the discovery and description of a new frog species from its urban core would evoke public interest and help to facilitate survey and conservation of amphibian biodiversity in Bangladesh.

## Materials and Methods

### Ethics statement

This study was conducted with appropriate permissions (CCF letter no. 22.01.0000.101.23.2012.681 for collecting specimens, CF memo no. 22.01.0000.101.23.2012 for transport) and guidelines from the responsible authority, the Forest Department, Ministry of Forest and Environment, the People’s Republic of Bangladesh. The protocols for our collection and research were approved by the committee of the Wildlife Section of the Forest Department, Bangladesh. Collected specimens were not recognized as belonging to threatened species and they are not listed in IUCN Redlist or by CITES.

### Morphological measurements and analyses

Measurements were taken with digital calipers to the nearest 0.02 mm. The 21 characters that were measured followed the definitions of Islam *et al*. [38] and Howlader *et al*. [39, 40]; their landmark definitions are depicted graphically in S1 Fig. The measurements included: SVL (distance from tip of snout to vent); HL (head length; distance from tip of snout to the back of mandible); HW (head width; maximum width of the head at the posterior margin of mandible); MN (distance from back of mandible to nostril); SL (snout length; distance from anterior corner of eye to the tip of snout); MFE (distance from back of mandible to front of the eye); MBE (distance from back of mandible to back of the eye); IN (internarial distance); IOD (interorbital distance); EN (distance from front of eyes to the nostril); NS (nostril—snout length); EL (eye width); UEW (maximum width of upper eyelid); HAL (hand length; distance from proximal end of outer palmar metacarpal tubercle to tip of third finger); FAL (forearm length; distance from corner of elbow to proximal end of outer palmar metacarpal tubercle); THIGHL (thigh length; distance from vent to knee); TL (tibia length; distance from knee to heel); TFOL (length of tarsus and foot; distance from heel to tip of fourth finger); FOL (foot length; distance from proximal end of inner metatarsal tubercle to tip of fourth finger). Webbing formula followed that of Glaw and Vences [41].

Apart from comparing ratios of different morphological traits between the new and other *Zakerana* species, we also performed discriminant function (DFA) and principal component analyses (PCA) to compare the new species (*Zakerana* sp. nov.) to its five (*viz*. *Z*. *asmati*, *Z*. *nepalensis*, *pierrei*, *Z*. *syhadrensis*, and *Z*. *teraiensis*) congeners for the 14 measurements (*viz*. SVL, HW, HL, SL, MBE, EN, NS, EL, IN, IOD, FAL, HAL, FOL, and TL) for which we had sufficient data. In the PCA, we extracted the two first axes, as they were the only ones with Eigenvalue > 1. Their scores were subjected to one-way ANOVA to formally compare if the mean scores differed among species. Tukey’s HSD post-hoc tests were used to examine if the mean values for the new species were significantly different from those of the other species.

Examined specimens are deposited at the Finnish Museum of Natural History, Finland (MZH), and Zoology Department, University of Chittagong, Bangladesh (MZD). Additional specimens used for morphological comparisons were examined from the collections of the Zoology Department, University of Chittagong, Bangladesh (MZD). Their accession numbers are listed in S1 Table.

### DNA analyses and phylogeny

Whole genomic DNA was extracted from muscle tissue (n = 3) using a silica-based method (Ivanova *et al*. [42]) and stored at -20°C. PCR amplification and sequencing of all samples was done with two pairs of primers (listed in S2 Table). PCR conditions for amplification of both genes consisted of 5.72 μl of dH_2_O, 2 μl of 5x buffer, 0.08 μl of dNTP, 0.2 μl of Phire enzyme (Thermo Fisher) and 0.5 μl of each primer, in a total reaction volume of 10 μl. The PCR program was comprised of a preliminary denaturation step at 98°C for 30 s, followed by 34 cycles of 98°C for 10 s, 55°C for 10 s, 72°C for 30 s and final extension at 72°C for 1 min. PCR products were purified by using ExoSap IT (USB Corporation, Cleveland, OH, USA) and sequenced at the Institute for Molecular Medicine, Finland (FIMM). Sequence ambiguities were edited by eye by aligning forward and reverse reads using the Geneious 5.6.5 program [43]. Obtained sequences were deposited in GenBank; accession numbers are provided in S3 Table.

The nucleotide sequences of the 16S and 12S rRNA genes were separately aligned with available sequences of *Zakerana* obtained from GenBank (n = 20, S3 Table) using the default parameters in ClustalW (built into BIOEDIT [44, 45]). From these two alignment datasets, sequence divergences (uncorrected *p*-values) were calculated using Mega v 5.5.6 [46] with the pairwise-deletion option, in which all aligned sites except indels were used for calibration. Phylogenetic analyses were conducted both with each gene fragment separately and also combined. The combined dataset was comprised of partial 16S (407 bp) and 12S (349 bp) gene sequences, resulting in a 756 bp sequence. Phylogenetic analyses were performed using Maximum likelihood (ML) and Bayesian inference methods. The most fitting nucleotide substitution model for the combined dataset was found to be the GTR + I + G substitution model. For the ML analysis, branch support was evaluated by using 1000 bootstrap replicates [47] as implemented in Mega v 5.5.6 [46]. For the Bayesian analyses, Markov chain Monte Carlo runs were performed for one million generations with a sampling frequency of 100, as implemented in MrBayes 3.1.2 [48]. Convergence of the runs was assessed from the average split frequency of standard deviations (<0.01) and by checking the potential scale reduction factors (~ 1.0) for all model parameters. 25% of the trees were discarded as burn-in and the remaining ones were used to generate a 50% majority rule consensus tree, as well as to estimate the Bayesian posterior probabilities.

### Bioacoustic analyses

**Fig 1:**
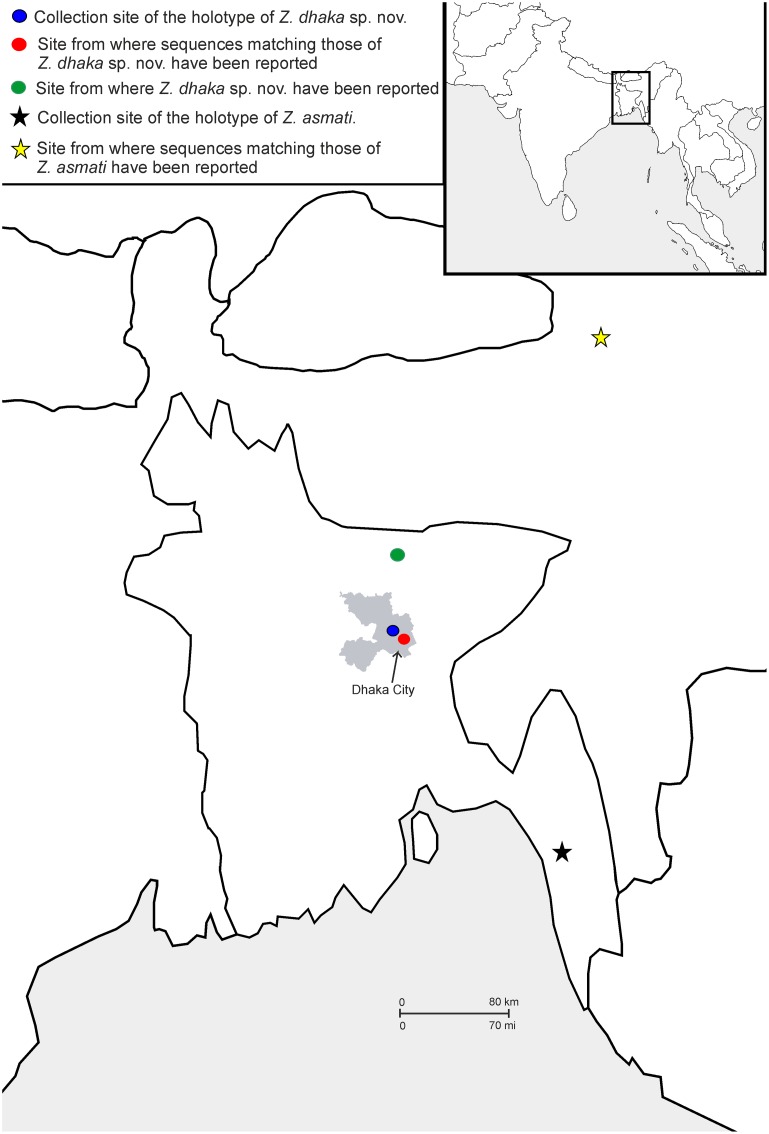
Maps showing the type locality for *Zakerana dhaka* sp. nov. found from Sher-e-Bangla Agricultural University campus, Sher-e-Bangla Nagar, Dhaka, Bangladesh (blue dot). The map also shows the other locality from where the new species was encountered within Dhaka, as well as the location from where individuals with the same haplotype have been encountered (see text for details). The type locality of *Zakerana asmati*, a species closely related to *Z*. *dhaka*, is indicated on the map with black star. The approximate borders of the urban core of Dhaka city are delineated in grey.

Call recordings were made using a Sony Cyber-shot camera on video mode (model: DSC-W530). During the recordings, the focal male was closer to the camera than to other calling neighbours. After locating an advertising male, advertisement calls were recorded from approx. 0.4 m distance. Advertisement calls of holotypes of both the new species and *Z*. *asmati* were recorded in air temperature between 23°C and 24°C when the individuals were calling on land in their respective type localities (Fig 1). Calls of *Z*. *asmati* were recorded in front of Teacher’s Club and Guest House (22°28'13.83”N, 91°47'36.06”E), Chittagong University campus, Hathazari, Chittagong, Bangladesh on 15 May 2008. The calls of the new species were recorded in front of the Kabi Kazi Nazrul Islam Hall (23°46'15.25"N, 90°22'39.87"E), Sher-e-Bangla Agricultural University campus, Sher-e-Bangla Nagar, Dhaka, Bangladesh on 11 June 2012.

Calls of the adult male (holotype) were analyzed with the acoustic software Adobe Audition 3.0 (following [49] and [50]), and compared to the described vocalizations of *Zakerana* species available in literature [34, 51–53]. Recordings were re-sampled at 44.1 Hz and 16 bit resolution in the mono pattern and in “Waveform” extension. Frequency information was obtained through Fast Fourier Transformation (FFT, width 1024 points); the audio spectrogram was obtained at Hamming window function with 256 bands resolution. We measured dominant frequency (the frequency in call containing the greatest energy, determined from Fourier transformation), pulse repetition rate (number of pulses repeated in a defined period of time within a note; the value is provided as pules per second), number of pulses per note (total number of pulses in a note), note repetition rate (number of notes repeated in a defined period of time within a call; the value is provided as notes per second), note duration (duration of temporally uninterrupted sound element composing the call and made up of pulses), inter-note intervals (the time elapsed from the end of a note to the beginning of the next note), as described by Cocroft and Ryan [54], Köhler [55], and Martins and Jim [56]. Mean, standard deviation and range (as well as number of analysed units, n), of call parameters with temporal measurements in seconds (s) or milliseconds (ms) are provided. Mann Whitney U-tests were used to determine if there are differences in call parameters between the new species and the phylogenetically closely related *Z*. *asmati*.

Statistical analyses were performed using R software 3.0.1 (The R Project for Statistical Computing, Vienna, Austria).

### Nomenclatural acts

The electronic edition of this article conforms to the requirements of the amended International Code of Zoological Nomenclature, and hence the new names contained herein are available under that Code from the electronic edition of this article. This published work and the nomenclatural acts it contains have been registered in ZooBank, the online registration system for the ICZN. The ZooBank LSIDs (Life Science Identifiers) can be resolved and the associated information viewed through any standard web browser by appending the LSID to the prefix "http://zoobank.org/". The LSID for this publication is: urn:lsid:zoobank.org:pub:C9DA37D3-EB61-4444-8C57-DB2E35843200. The electronic edition of this work was published in a journal with an ISSN, and has been archived and is available from the following digital repositories: PubMed Central, LOCKSS.

## Results

### Taxonomic treatment

Amphibia, Linnaeus, 1758

Anura Fischer von Waldheim, 1813

Dicroglossidae Anderson, 1871

Dicroglossinae Anderson, 1871

*Zakerana* Howlader, 2011

*Zakerana dhaka* sp. nov. urn:lsid:zoobank.org:act:64954E38-8611-4067-994F-820B7F787A6A

#### Etymology

The species name derives from the name of the type locality, Dhaka city, from where the type specimens were collected. The specific epithet is treated as a noun in apposition.

#### Holotype

Adult male, MZH-3371, collected in front of Kabi Kazi Nazrul Islam Hall (23°46'15.25"N, 90°22'39.87"E), Sher-e-Bangla Agricultural University campus, Sher-e-Bangla Nagar, Dhaka, Bangladesh; collected by M. S. A. Howlader, June 11, 2012.

#### Paratopotypes (n = 11)

MZH-3372 (adult female), MZH-3373 (adult male), MZD-1031 (adult male), MZD-1032 (adult male), MZD-1033 (adult male), MZD-1034 (adult male), MZD-1035 (adult male), MZD-1036 (adult male), MZD-1037 (adult male), MZD-1038 (adult female), and MZD-1039 (adult female). All specimens were collected from the same locality as the holotype on June 10, 2012 and May 21, 2013 by M. S. A. Howlader, N. Ahsan and R. Tapu.

#### Diagnosis

**Fig 2:**
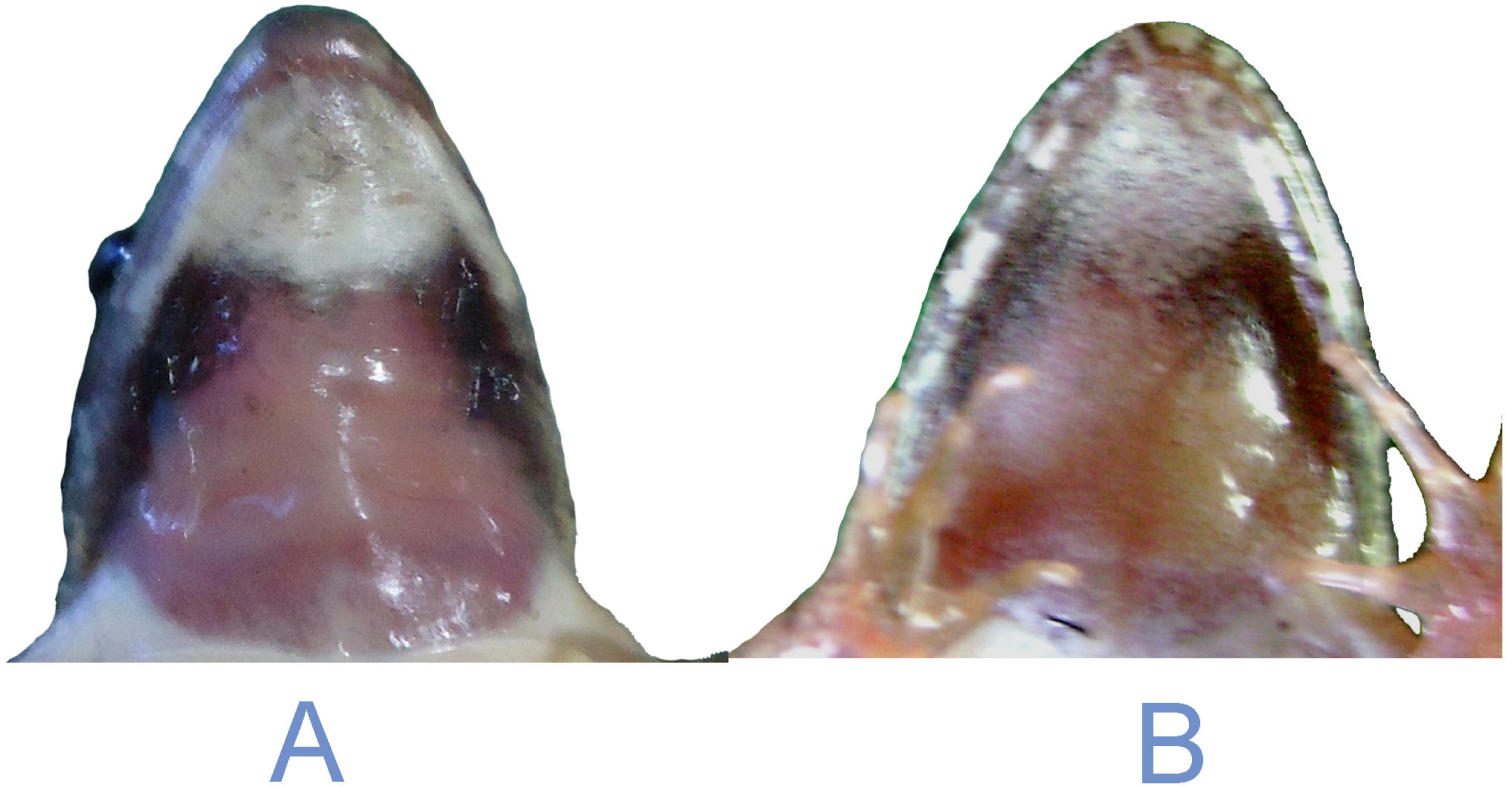
Photographs of ventral view of snout in (A) *Zakerana dhaka* sp. nov. and (B) *Zakerana asmati*.

The species is assigned to the genus *Zakerana* by having small body size, pointed snout, relatively small tympanum, small inner metatarsal tubercle, rudimentary webbing on feet, “Fejervaryan” line on both sides of belly, and by its phylogenetic positioning in molecular analyses. *Zakerana dhaka* sp. nov. differs from its congeners occurring in Bangladesh as follows (Table 1, S4 Table): trapezoid-shaped fleshy red vocal sac markings on throat in males (*vs*. butterfly shaped in *Z*. *asmati*; laterally dark and medially pale in both *Z*. *nepalensis* and *Z*. *pierrei*; crescent shaped in *Z*. *syhadrensis*; “W” shaped in *Z*. *teraiensis*); forearm length 52–58% of hand length (*vs*. forearm length 70–96% of hand length in *Z*. *asmati*, *Z*. *nepalensis*, and *Z*. *pierrei*; forearm length equal to hand length in both *Z*. *syhadrensis* and *Z*. *teraiensis*); relative length of fingers: 2 < 4 < 1 < 3 (*vs*. relative length of fingers: 2 = 4 < 1 < 3 in both *Z*. *pierrei* and *Z*. *teraiensis*; 2 < 1 < 4 < 3 in *Z*. *nepalensis*; 1 = 2 < 4 < 3 in *Z*. *syhadrensis*; but similar in *Z*. *asmati*); foot length 81–86% of tibia length (*vs*. foot length is equal or nearly equal to tibia length in *Z*. *asmati*, *Z*. *nepalensis*, *Z*. *pierrei*, *Z*. *syhadrensis*, and *Z*. *teraiensis*); internarial distance 1.58–1.73 times greater than distance from nostril to snout tip (*vs*. < 1.5 times in *Z*. *asmati*, *Z*. *nepalensis*, and *Z*. *syhadrensis*; approximately equal in *Z*. *pierrei* and *Z*. *teraiensis*). Interorbital distance 62–68% of internarial distance in male (*vs*. 73–95% in *Z*. *asmati*, *Z*. *nepalensis*, *Z*. *pierrei*, *Z*. *syhadrensis*, and *Z*. *teraiensis*). Moreover, *Z*. *dhaka* sp. nov. differs distinctly from closely related *Z*. *asmati* in having a flattened snout which projects over the lower jaw (vs. rounded snout not much projected over lower jaw in *Z*. *asmati*) in a ventral view (Fig 2).

**Table 1:** Summary of quantitative and qualitative diagnostic characters in *Zakerana dhaka* sp. nov. and its closest congeners. Morphological ratios given as mean (± S.D.) over range.

	*Z*. *dhaka* sp. nov.		*Z*. *asmati*		*Z*. *nepalensis*		*Z*. *pierrei*		*Z*. *syhadrensis*		*Z*. *teraiensis*	
	Male n = 9	Female n = 3	Male n = 5	Female n = 1	Male n = 5	Female n = 6	Male n = 2	Female n = 3	Male n = 6	Female n = 3	Male n = 10	Female n = 11
**HL:SVL**	0.35±0.01 (0.34–0.37)	0.35±0.01 (0.34–0.36)	0.34±0.01 (0.32–0.36)	0.34	0.34±0.01 (0.32–0.36)	0.34±0.01 (0.33–0.37)	0.31 (0.30–0.33)	0.33±0.00 (0.32–0.33)	0.32±0.00 (0.32–0.36)	0.33±0.01 (0.32–0.35)	0.33±0.01 (0.31–0.36)	0.33±0.01 (0.31–0.34)
**HL:HW**	1.03±0.01 (1.03–1.06)	1.04±0.01 (1.03–1.06)	1.07±0.01 (1.10–1.05)	1.08	1.06±0.02 (0.98–1.02)	1.02±0.03 (0.99–1.05)	1.02 (1.01–1.03)	1.06±0.02 (1.03–1.08)	1.03±0.03 (0.99–1.09)	1.04±0.03 (1.03–1.08)	1.00±0.01 (0.96–1.03)	1.03±0.03 (0.95–1.06)
**SL:HL**	0.40±0.01 (0.40–0.43)	0.39±0.01 (0.38–0.40)	0.38±0.01 (0.37–0.40)	0.40	0.45±0.04 (0.39–0.49)	0.39±0.04 (0.34–0.44)	0.43 (0.42–0.44)	0.43±0.03 (0.40–0.47)	0.47±0.03 (0.42–0.51)	0.45±0.06 (0.40–0.51)	0.47±0.02 (0.44–0.49)	0.43±0.02 (0.40–0.46)
**EN:NS**	1.23±0.04 (1.20–1.29)	1.27±0.02 (1.24–1.28)	1.77±0.04 (1.74–1.80)	1.66	1.24±0.12 (1.05–1.35)	1.45±0.19 (1.13–1.64)	1.18(1.13–1.24)	1.13±0.07 (1.05–1.20)	1.14±0.16 (0.92–1.41)	1.22±0.15 (1.05–1.35)	1.03±0.07 (0.91–1.13)	1.06±0.11 (0.93–1.32)
**IN:NS**	1.59±0.01 (1.58–1.60)	1.70±0.02 (1.69–1.73)	1.49±0.03 (1.45–1.51)	1.43	1.33±0.10 (1.15–1.39)	1.54±0.14 (1.31–1.73)	1.18 (1.18–1.19)	1.23±0.09 (1.15–1.33)	1.38±0.21 (1.18–1.76)	1.44±0.12 (1.30–1.52)	0.99±0.09 (0.76–1.09)	1.10±0.09 (0.95–1.31)
**EL:HL**	0.41±0.01 (0.40–0.43)	0.41±0.01 (0.40–0.42)	0.42±0.01 (0.40–0.43)	0.41	0.37±0.03 (0.32–0.40)	0.35±0.03 (0.30–0.39)	0.34 (0.34–0.34)	0.36±0.04 (0.31–0.39)	0.37±0.02 (0.33–0.40)	0.33±0.04 (0.29–0.37)	0.34±0.01 (0.32–0.37)	0.32±0.01 (0.29–0.35)
**EL:SVL**	0.14±0.01 (0.13–0.17)	0.14±0.01 (0.13–0.15)	0.15±0.01 (0.14–0.16)	0.14	0.13±0.01 (0.11–0.15)	0.12±0.01 (0.10–0.13)	0.11 (0.10–0.11)	0.12±0.01 (0.10–0.13)	0.12±0.00 (0.11–0.13)	0.11±0.01 (0.10–0.12)	0.11±0.00 (0.10–0.12)	0.11±0.00 (0.09–0.12)
**IOD:IN**	0.63±0.01 (0.62–0.65)	0.65±0.03 (0.63–0.68)	0.88±0.03 (0.86–0.90)	0.88	0.92±0.07 (0.81–1.00)	0.91±0.11 (0.71–1.00)	0.95(0.95–0.95)	0.83±0.03 (0.80–0.87)	0.75±0.13 (0.61–0.96)	0.63±0.07 (0.55–0.69)	0.77±0.10 (0.55–0.90)	0.64±0.08 (0.53–0.78)
**MBE:HL**	0.23±0.01 (0.22–0.25)	0.23±0.02 (0.22–0.25)	0.18±0.01 (0.17–0.19)	0.19	0.29±0.03 (0.27–0.32)	0.28±0.03 (0.24–0.32)	0.30 (0.30–0.31)	0.31±0.01 (0.30–0.32)	0.31±0.03 (0.27–0.35)	0.33±0.04 (0.29–0.38)	0.33±0.03 (0.27–0.38)	0.34±0.05 (0.28–0.41)
**FAL:HAL**	0.54±0.02 (0.52–0.58)	0.54±0.02 (0.53–0.55)	0.71±0.03 (0.69–0.73)	0.75	0.85±0.08 (0.80–0.96)	0.87±0.09 (0.79–0.95)	0.86 (0.85–0.86)	0.83±0.02 (0.80–0.84)	1.00±0.01 (0.96–1.02)	0.97±0.02 (0.95–1.00)	1.01±0.07 (0.92–1.21)	1.02±0.09 (0.93–1.27)
**FOL:TL**	0.83±0.02 (0.81–0.86)	0.82±0.02 (0.81–0.83)	1.04±0.01 (1.03–1.05)	1.04	1.01±0.02 (0.97–1.03)	0.97±0.01 (0.92–1.03)	0.99 (0.99–0.99)	1.06±0.08 (0.99–1.15)	1.01±0.08 (0.85–1.08)	1.00±0.03 (0.97–1.03)	0.97±0.05 (0.90–1.07)	0.94±0.06 (0.87–1.00)
**Vocal sac marking on throat**	Trapezoid shaped fleshy red color marking with black side line	----	Butterfly shaped dark marking	----	Laterally dark and medially pale	----	Laterally dark and medially pale	----	Crescent shaped dark marking	----	“W” shaped dark marking	----
**Relative length of fingers**	2<4<1<3	2<4<1<3	2<4<1<3	2<4<1<3	2<1<4<3	2<1<4<3	2 = 4<1<3	2 = 4<1<3	1 = 2<4<3	1 = 2<4<3	2 = 4<1<3	2 = 4<1<3
**Middorsal line**	Distinct & narrow	Absent or present	Distinct & narrow	Distinct & narrow	Distinct & narrow	Distinct & narrow	Distinct broad with same width throughout from snout to vent	Distinct with same width throughout from snout to vent	Absent	Absent	Variable	Variable
**Skin fringe on outer side of 5**^**th**^ **toe**	Weak and indistinct	Weak and indistinct	Weak and indistinct	Weak and indistinct	Very indistinct	Very indistinct	Absent	Absent	Vestige or reduced	Vestige or reduced	Distinct	Distinct
**Body size (SVL)**	27.81–31.59 mm	33.48–37.93 mm	29.1–30.0 mm	33.4 mm	21.4–30.1 mm	22.5–31.2 mm	25.6–27.8 mm	23.1–29.4 mm	27.1–30.0 mm	32.8–37.2 mm	37.8–44.1 mm	35.7–52.3 mm

*Z*. *dhaka* sp. nov. can be further distinguished from *Z*. *asmati*, *Z*. *pierrei*, *and Z*. *teraiensis* by the following additional criteria: distance from anterior margin of eye to the nostril is 1.20–1.29 times greater than distance from nostril to snout tip (*vs*. 1.66–1.77 in *Z*. *asmati*; and 1.03–1.18 in *Z*. *pierrei* and *Z*. *teraiensis*); snout length 38–40% of head length (*vs*. snout length 40–51% of length in *Z*. *asmati*, *Z*. *pierrei*, *Z*. *syhadrensis*, and *Z*. *teraiensis*); middorsal line is narrow and distinct (*vs*. distinct but very wide all the way from snout to vent in *Z*. *pierrei*, absent in *Z*. *syhadrensis*, present or absent in *Z*. *teraiensis*); two red spots found on the middorsal line and additional two red spots on each of the forelimbs are usually present in males (none of the other species, except for *Z*. *asmati*, has these spots).

**Fig 3:**
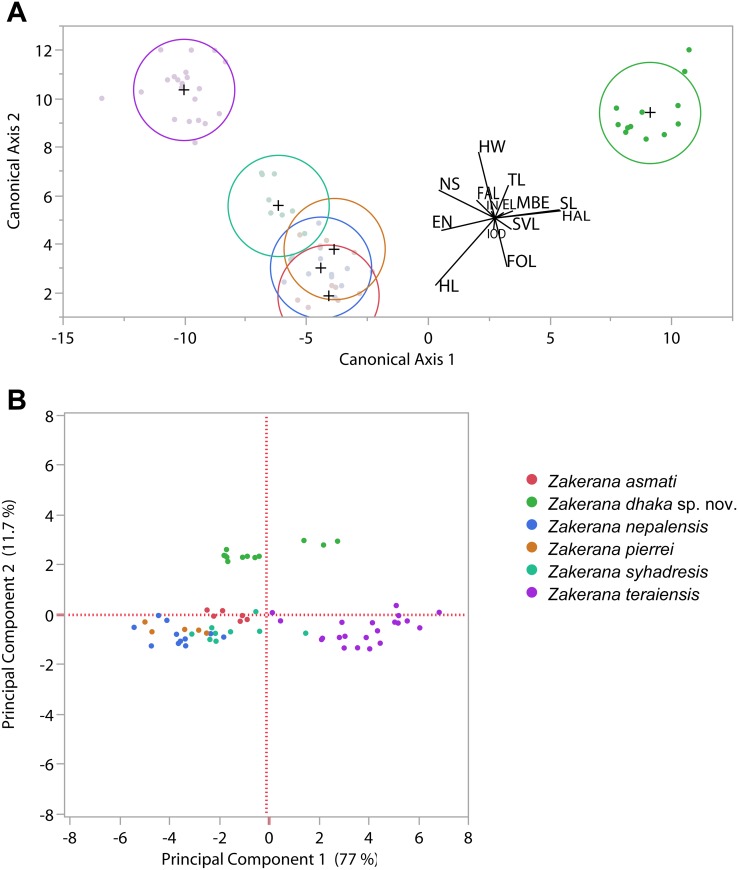
Results of the multivariate analyses of morphometric variability in *Zakerana dhaka* sp. nov., *Z*. *asmati*, *Z*. *pierrei*, *Z*. *nepalensis*, *Z*. *syhadrensis*, and *Z*. *teraiensis*. (A) Discriminant function and (B) principal component analyses of the data.

Formal multivariate analyses supported the inference based on the comparisons above: DFA of 14 morphometric traits identified *Z*. *dhaka* sp. nov. as clearly different from the other species (Fig 3A). Likewise, PCA showed that while *Z*. *dhaka* sp. nov. exhibited intermediate values on the first PCA-axis that were not significantly different from *Z*. *asmati* and *Z*. *pierrei* (Tukey’s HSD, p > 0.05), it was clearly distinct from all other species along the second axis (Tukey’s HSD, p < 0.05; Fig 3B). Close inspection of the matrix loadings revealed that the first axis was a general size factor (S5 Table) with all traits loading strongly and positively, and the second axis captured shape differences mainly attributable to effects of traits HAL and IOD, but to some degree also to those of TL, MBE, EL and EN (S5 Table).

**Fig 4:**
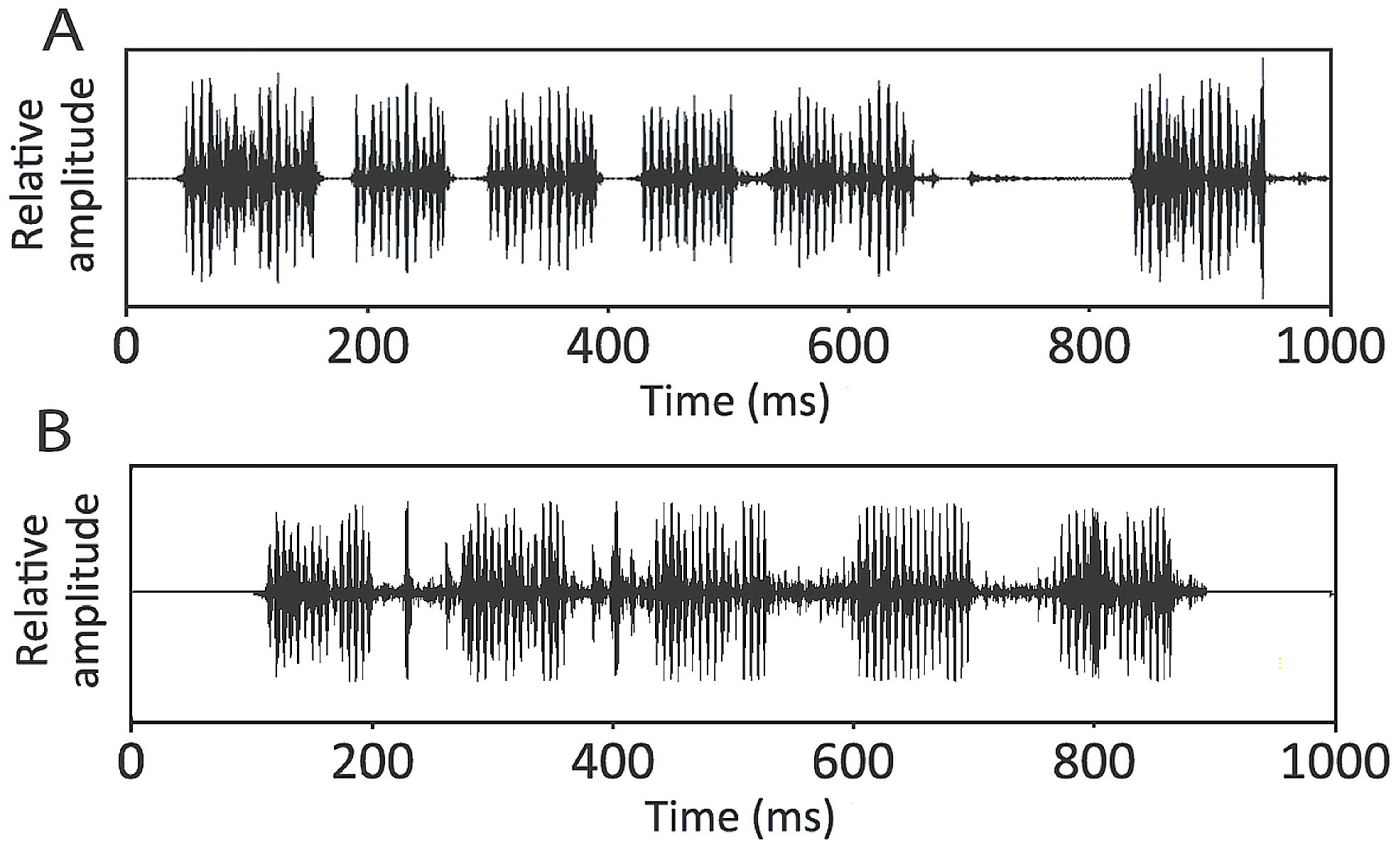
Waveforms of (A) *Zakerana dhaka* sp. nov. and (B) *Zakerana asmati* advertisement calls. Relative amplitudes were roughly similar between the two species.

We compared the advertisement call of *Z*. *dhaka* with call of *Z*. *asmati*, the most closely related species to *Z*. *dhaka* ([34]; Fig 4) recorded at the same temperature. Both calls present similarities but differ in two parameters: *Z*. *asmati* has a greater duration of inter-note intervals than *Z*. *dhaka* (W = 550; p = 0.001; *Z*. *asmati*, *x* = 56.25 ± 12.63, n = 4; *Z*. *dhaka*, *x* = 32.4 ± 6.7, n = 140) and shows a significantly different dominant frequency (W = 458; p < 0.05, Fig 4; *Z*. *asmati*: 4100–5100 Hz; *Z*. *dhaka*: 2600–3800 Hz). The two species do not differ in their note duration (W = 455; p = 0.93; *Z*. *asmati*: 17–134 ms; *Z*. *dhaka*: 61–144 ms) and number of pulses per note (W = 672, p = 0.11; *Z*. *asmati*, *x* = 16.8 ± 1.3, n = 5; *Z*. *dhaka*, *x* = 14.5 ± 3.2, n = 190).

According to the original advertisement call descriptions of *Z*. *nepalensis*, *Z*. *pierrei*, *Z*. *teraiensis* [51–53], and *Z*. *syhadrensis* [53], *Z*. *dhaka* is significantly different in various call properties. The average pulse repetition rate in *Z*. *dhaka* was 144.5 ± 4.2 pulses/s (n = 8) [vs. 76.4 (n = 1) pulses/s in *Z*. *pierrei*, 199.7 ± 8.8 (n = 28) pulses/s in *Z*. *syhadrensis*, 74.8 pulses/s (n = 1) in *Z*. *teraiensis*], average number of pulses per note was 14.5 ± 3.2 (n = 190) [vs. 58.2 ± 2.8 (n = 5) in *Z*. *nepalensis*, 19.7 ± 1.9 (n = 9) in *Z*. *pierrei*, 5.3 ± 0.5 (n = 9) in *Z*. *teraiensis*], note repetition rate was about 5.7–6.6/s [vs. 1.3–2.2 /s in *Z*. *nepalensis*, 2.8–3.2 in *Z*. *pierrei*, 9.1–9.3 in *Z*. *syhadrensis*, 2.8–3.6 in *Z*. *teraiensis*], duration of notes lasted between 0.061 to 0.144 s [vs. 0.164 to 0.406 in *Z*. *nepalensis* and *Z*. *pierrei*], inter-note intervals ranged from 0.018 to 0.05 s [vs. 0.158 to 0.505 s in *Z*. *nepalensis* and *Z*. *teraiensis*, 0.072 to 0.274 in *Z*. *pierrei*].

*Zakerana dhaka* sp. nov. can be distinguished from other south Asian species of *Zakerana* (15 species) by the following characters (Table 2): relative finger length is 2 < 4 < 1 < 3 (*vs*. no other south Asian species have this character except *Z*. *mysorensis*), head is longer than wide (*vs*. head is wider than long in *Z*. *brevipalmata*, *Z*. *caperata*, *Z*. *granosa*, *Z*. *greenii*, *Z*. *keralensis*, *Z*. *kudremukhensis*, *Z*. *mudduraja*, *Z*. *mysorensis*, *Z*. *nilagirica*, *Z*. *rufescens*, and *Z*. *sengupti*), having a distinct outer metatarsal tubercle (*vs*. outer metatarsal tubercle indistinct in *Z*. *brevipalmata*, *Z*. *greenii*, and *Z*. *parambikulamana*), interorbital distance is less than maximum width of upper eyelid (*vs*. maximum width of upper eyelid is greater than interorbital distance in both *Z*. *kirtisinghei* and *Z*. *sauriceps*), IOD 74% of UEW [vs. IOD is equal or more than 90% of UEW in *Z*. *caperata* (n = 35), *Z*. *granosa* (n = 33) and *Z*. *kudremukhensis* (n = 22); IOD is 83% of UEW in *Z*. *nilagirica* (n = 10); IOD is above 50% of UEW in *Z*. *keralensis* (n = 10) and *Z*. *rufescens* (n = 13); IOD is above 60% of UEW in *Z*. *brevipalmata* (n = 9), *Z*. *mudduraja* (n = 12), *Z*. *parambikulamana* (n = 1) and *Z*. *sengupti* (n = 5); IOD is 40% of UEW in *Z*. *sauriceps* (n = 1)], nostril—snout length is greater than distance from front of eyes to the nostril (*vs*. distance from front of eyes to the nostril is equal or greater than nostril—snout length in *Z*. *brevipalmata*, *Z*. *greenii*, *Z*. *nilagirica*, *Z*. *parambikulamana*, *Z*. *rufescens*, and *Z*. *sengupti*), NS 78% of EN [vs. NS is more than 90% of EN in *Z*. *caperata* (n = 35), *Z*. *granosa* (n = 33), *Z*. *kudremukhensis* (n = 22), and *Z*. *keralensis* (n = 10)], IOD 64% of IN [vs. IOD is equal of IN in both of *Z*. *mysorensis* and *Z*. *sauriceps*; IOD is more than 80% of IN in both *Z*. *caperata* (n = 35) and *Z*. *granosa* (n = 33); IOD is half of IN in *Z*. *rufescens*; IOD is above or more than 74% of IN in *Z*. *kudremukhensis* (n = 22), *Z*. *nilagirica* (n = 10) and *Z*. *sengupti* (n = 5)], HAL 46% of SVL (vs. HAL is above or less than 25% of SVL in other known south Asian species; Table 2), tibia length is more than half of SVL (vs. tibia length is half or less than half of SVL in *Z*. *brevipalmata*, *Z*. *caperata*, *Z*. *mudduraja*, *Z*. *parambikulamana*, *Z*. *rufescens*, and *Z*. *sauriceps*), there is no pit behind the snout tip (vs. pit is present in *Z*. *sauriceps*), reduced webbing between toes (vs. developed webbing present between toes in *Z*. *brevipalmata*, *Z*. *greenii*, *Z*. *keralensis*, *Z*. *kirtisinghei*, *Z*. *mysorensis*, and *Z*. *sauriceps*). Pillai [28] diagnosed *Z*. *murthii* (as *Rana murthii*) with the most diagnostic feature of this species being the presence of two triangular patches bearing pearl-like papillae on the breast in males, and also the presence of the papillae in the anterior part of the lower jaw. We, however, could not find such papillae in the new species.

**Table 2:** A comparison of *Zakerana dhaka* sp. nov. with its congeners found in south Asia (except Bangladesh). M = matched with the character; NM = not matched with the character; “---” = data not available.

	Head longer than wide	Outer metatarsal tubercle distinct	Relative lengths of fingers	IOD/UEW (“%” Mean)	EN/NS (“%” Mean)	IOD/IN (“%” Mean)	TL/SVL (“%” Mean)	HAL/SVL (“%” Mean)	Pit behind the snout tip	Webbing between the toes	Uninterrupted or regular glandular folds along the back	Coloration and distinct characters	References
***Z*. *dhaka* sp. nov**.	M	M	2<4<1<3	IOD 74% of UEW (n = 12)	NS 78% of EN (n = 12)	IOD 64% of IN (n = 12)	TL 65% of SVL (n = 12)	HAL 46% of SVL (n = 12)	NM	Reduced	NM	Trapezoid shaped fleshy red color vocal sac marking present in male	Present study
***Z*. *brevipalmata***	NM	NM	4<2<1<3	IOD 67% of UEW(n = 9)	EN 89% of NS (n = 9)	IOD 63% of IN (n = 9)	TL 52% of SVL (n = 9)	HAL 23% of SVL (n = 9)	NM	Partially	NM	Laterally dark and medially pale vocal marking	Kuramoto *et al*. [26]; Boulenger [20]
***Z*. *caperata***	NM	---	4<2<1<3	IOD 90% of UEW (n = 35)	NS 89% of EN (n = 35)	IOD 87% of IN (n = 35)	TL 50% of SVL (n = 35)	HAL 23% of SVL (n = 35)	NM	Reduced	NM	Mid-dorsal stripe broad, pale	Kuramoto *et al*. [26]
***Z*. *granosa***	NM	---	4<2<1<3	IOD 96% of UEW (n = 33)	NS 94% of EN (n = 33)	IOD 82% of IN (n = 33)	TL 54% of SVL (n = 33)	HAL 24% of SVL (n = 33)	NM	Reduced	NM	Dorsum with a narrow mid-dorsal stripe; round dark-red marking on dorsum at level of forelimbs	Kuramoto *et al*. [26]
***Z*. *greenii***	NM	NM	4<1 = 2<3	---	NS equal of EN (n = 8)	---	TL half of SVL (n = 8)	---	NM	Entirely	M	Brown above, with black spots on the body	Boulenger [20, 21]
***Z*. *keralensis***	NM	---	2<1	IOD 53% of UEW (n = 10)	NS 97% of EN (n = 10)	IOD 68% of IN (n = 10)	TL 56% of SVL (n = 10)	HAL 24% of SVL (n = 10)	NM	Entirely	NM	Grey or brown above, darker spotted; male has two internal vocal sacs	Kuramoto *et al*. [26]
***Z*. *kirtisinghei***	M	M	2<1<4<3	UEW 96% of IOD (n = 9)	---	---	TL 55% of SVL (n = 9)	---	NM	Entirely	M	Ventrally pale yellow	(Manamendra-Arachchi and Gabadage [27]
***Z*. *kudremukhensis***	NM	M	4<2<1<3	IOD 93% of UEW (n = 22)	NS 95% of EN (n = 22)	IOD 74% of IN (n = 22)	TL 58% of SVL (n = 22)	HAL 22% of SVL (n = 22)	NM	Reduced	NM	Pale middorsal stripe from snout to vent, widening behind inverse V-shaped ridge	Kuramoto *et al*. [26]
***Z*. *mudduraja***	NM	M	4<2<1<3	IOD 60% of UEW (n = 12)	NS 80% of EN (n = 12)	IOD 61% of IN (n = 12)	TL 49% of SVL (n = 12)	HAL 25% of SVL (n = 12)	NM	Reduced	NM	Dark inverse V-shaped marking inside interrupted inverse V-shaped ridge	Kuramoto *et al*. [26]
***Z*. *mysorensis***	NM	M	2<4<1<3	---	---	IOD equal of IN (n = 1)	TL 55% of SVL (n = 1)	---	NM	Entirely	M	V shaped dark band between the eyes	Rao [30]
***Z*. *nilagirica***	NM	---	2 = 4<1<3	IOD 83% of UEW (n = 10)	EN 96% of NS (n = 10)	IOD 77% of IN (n = 10)	TL 54% of SVL (n = 10)	HAL 24% of SVL (n = 10)	NM	Reduced	NM	---	Jerdon [33]; Kuramoto *et al*. [26]; Boulenger [20]
***Z*. *parambikulamana***	M	NM	3<4<2<1	IOD 67% of UEW (n = 1)	EN 83% of NS (n = 1)	IOD 67% of IN (n = 1)	TL 46% of SVL (n = 1)	HAL 23% of SVL (n = 1)	NM	Reduced	M	Yellowish brown above, ventral surface white	Rao [31]
***Z*. *rufescens***	NM	M	2 = 4<1<3	IOD 50% of UEW (n = 13)	EN equal of NS (n = 13)	IOD 50% of IN (n = 13)	TL 51% of SVL (n = 13)	HAL 24% of SVL (n = 13)	NM	Moderate	NM	“W” shaped dark vocal marking	Jerdon [33]; Kuramoto *et al*. [26]; Boulenger [20]
***Z*. *sauriceps***	M	---	1 = 2<4<3	UEW 40% of IOD (n = 1)	NS 75% of EN (n = 1)	IOD equal of IN (n = 1)	TL 40% of SVL (n = 1)	HAL 16% of SVL (n = 1)	M	Partially	NM	Above chocolate red, the undersurface of the thighs pale orange	Rao [31]
***Z*. *sengupti***	NM	M	2<1<4<3	IOD 64% of UEW (n = 5)	EN 90% of NS (n = 5)	IOD 74% of IN (n = 5)	SVL 61% of TL (n = 5)	---	NM	Moderate	NM	Forearm with prominent red spots	Purkayastha and Matsui [29]

### Description of the holotype (adult male)

Small sized frog (SVL 30.46 mm). Head large, triangular, longer than wide, HW 96% of HL, HW 35% of SVL, HL 36% of SVL, MFE 67% of HL, MBE 25% of HL. Snout flattened from ventral view, snout projected over lower jaw, SL 40% of HL; canthus rostralis indistinct, loreal region concave. Nostrils much closer to snout tip than to eyes, NS 78% of EN; NS 6% of SVL, EN 7% of SVL; nostrils rounded and very small, NS 63% of IN, MN 84% of HL. Eye large, EL 42% of HL, EL 15% of SVL; maximum width of upper eyelid greater than interorbital distance, IOD 74% of UEW, UEW 52% of EL, UEW 8% of SVL. Interorbital space convex, IOD 64% of IN. Tympanum round, TD 43% of EL.

Arms moderately long, robust, FAL 55% of HAL, FAL 25% of SVL, HAL 46% of SVL. Fingers small, free of webbing, tips rounded. Relative length of fingers, 2 < 4 <1 < 3; tips of fingers bluntly rounded; fingers lacking dermal ridge. Subarticular tubercles prominent, rounded, a single tubercle per digit; supernumerary tubercles absent; two oval shaped, distinct palmar tubercles.

**Fig 5:**
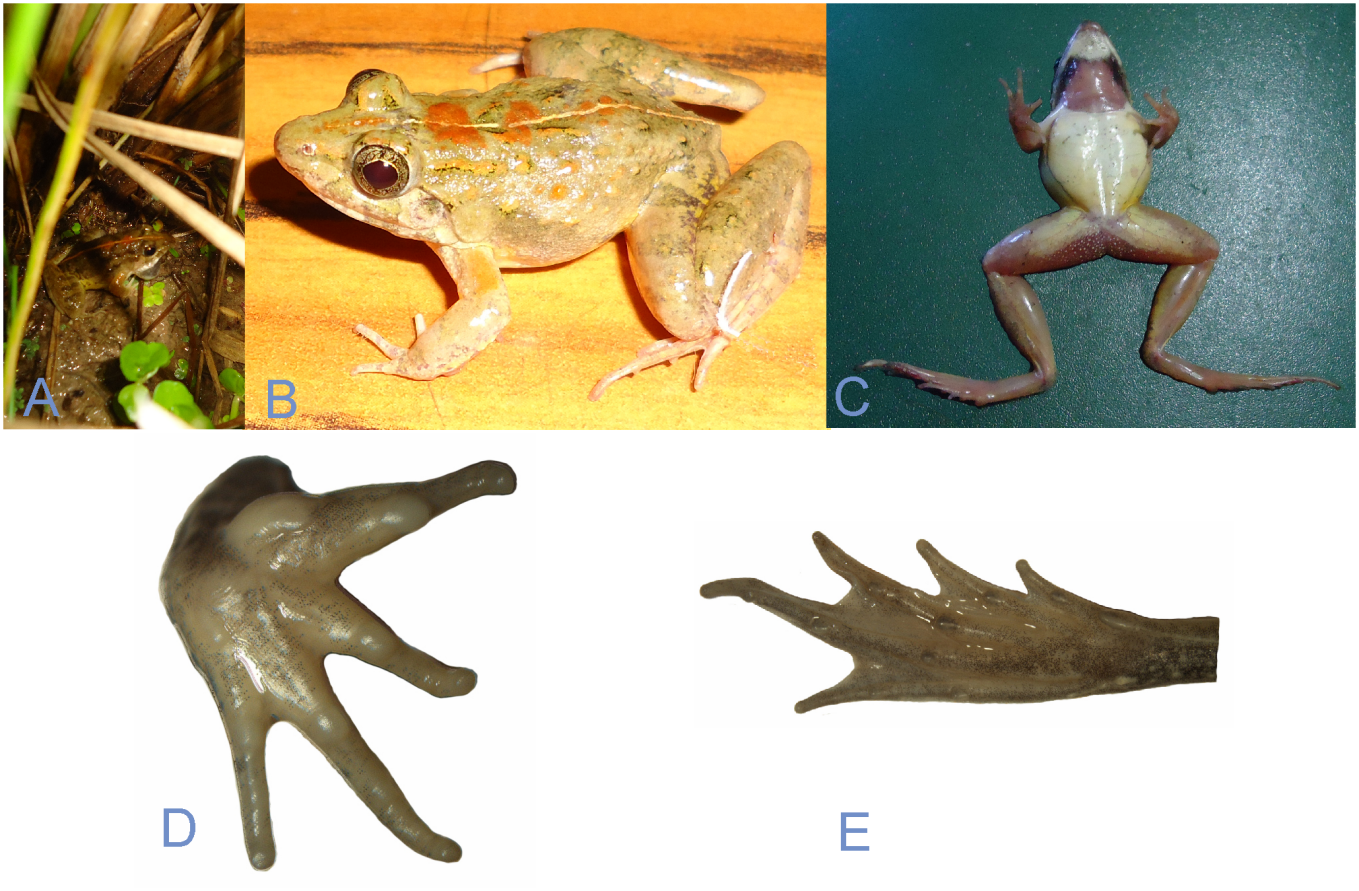
Photographs of *Zakerana dhaka* sp. nov. (A & B) Dorso-lateral view of male (holotype, live), (C) ventral view, ventral view of (D) hand and (E) foot.

Hind limbs relatively long, TL 65% of SVL, THIGHL 85% of TL; FOL 54% of SVL and FOL 84% of TL, FOL 66% of TFOL. Toes long, thin, tips rounded; webbing between toes weakly developed [1(1), 2i (1.5), 2e (0.5), 3i (2), 3e (1), 4i (2), 4e (2.5), 5 (0.75)]. Relative lengths of toes, 1 < 2 < 5 < 3 < 4; a weak, indistinct fringe of skin on outer side of toe 5. Inner metatarsal tubercle elongated, present at base of toe 1; outer metatarsal tubercle is oval, minute, distinct; subarticular tubercles well-developed, nearly oval. Dorsal surface of body smooth, tubercles present, arranged in row or reticulated pattern; tiny granules on upper eyelids, loreal, and cloacal region (Fig 5). Dorsal surface of forelimbs, thigh and tarsus glandular. Throat, chest, abdomen and ventral part of thigh and tibia smooth.

#### Coloration in life

Basic color of dorsal surface olive green with a few dark irregular spots fused into transverse bands; spots are formed by tubercle arrangement on dorsal side; sides of the body marbled brown. A narrow mid-dorsal line present from vent up to the posterior of eyes. Two red spots are found on the mid-dorsal line. Two additional red spots are present on each of the forelimbs. Toe webbings faintly marbled. Forelimbs with dark transverse bands; hind limbs with pigmented spots on thighs and stripes on lower legs. Ventral surface immaculate.

#### Coloration in preservative

In preservative, general color pattern remained unaffected, but colors faded. Olive green surfaces became whitish brown. Mid-dorsal line and forelimbs lost their red spots.

#### Measurements (in mm)

Male (holotype): SVL 30.46; HL11.06; HW 10.67; MN 9.35; SL 4.42; MFE 7.37; MBE 2.77; IN 2.84; IOD 1.81; EN 2.29; NS 1.79; EL 4.63; UEW 2.43; TD 2.01; HAL 13.90; FAL 7.61; LAL 6.73; THIGHL 16.85; TL 19.72; TFOL 24.85; FOL 16.55. Female (paratopotype; MZH-3372): SVL 37.48; HL 12.61; HW 11.88; MN 10.43; SL 4.79; MFE 8.39; MBE 2.81; IN 3. 55; IOD 2.40; EN 2.62; NS 2.05; EL 5.24; UEW 3.25; TD 2.41; HAL 16.87; FAL 9.20; LAL 7.98; THIGHL 20.40; TL 24.29; TFOL31.52; FOL 19.86.

#### Variation in paratopotypes

Accession numbers and morphometric variability are shown in Table 1 and S4 Table. One specimen lacked a mid-dorsal line (male MZH-3372). Female (MZH-3373) had no vocal sac markings and lacked red spots. In regard to coloration in life and preservative, the descriptions of the holotype apply: there was little variation among individuals in the type series.

#### Distribution

*Zakerana dhaka* is thus far known only from Dhaka, Bangladesh, from the type locality and Curzon Hall (23°43'34.67"N, 90°24'4.91"E) situated 5.6 km from the type locality (Fig 1). However, the species also seems to be present in areas adjacent to Dhaka city, such as Mymenshing district (Fig 1) from where two unidentified specimens with haplotypes similar to that of the new species have been reported [38,57]. In comparison, the map (Fig 1) also shows the localities from where the species *Z*. *asmati* has reported.

#### Natural history

The new species was observed only at night in the rain. At the type locality, specimens were found in or around temporary pools located in a grass field. In the Curzon Hall site, the species was found in an unused sewage canal of a building. Hence, the new species seems to be resistant to urban disturbance by occupying small habitat patches in different places that are subject to human commotion.

#### Advertisement call

**Fig 6:**
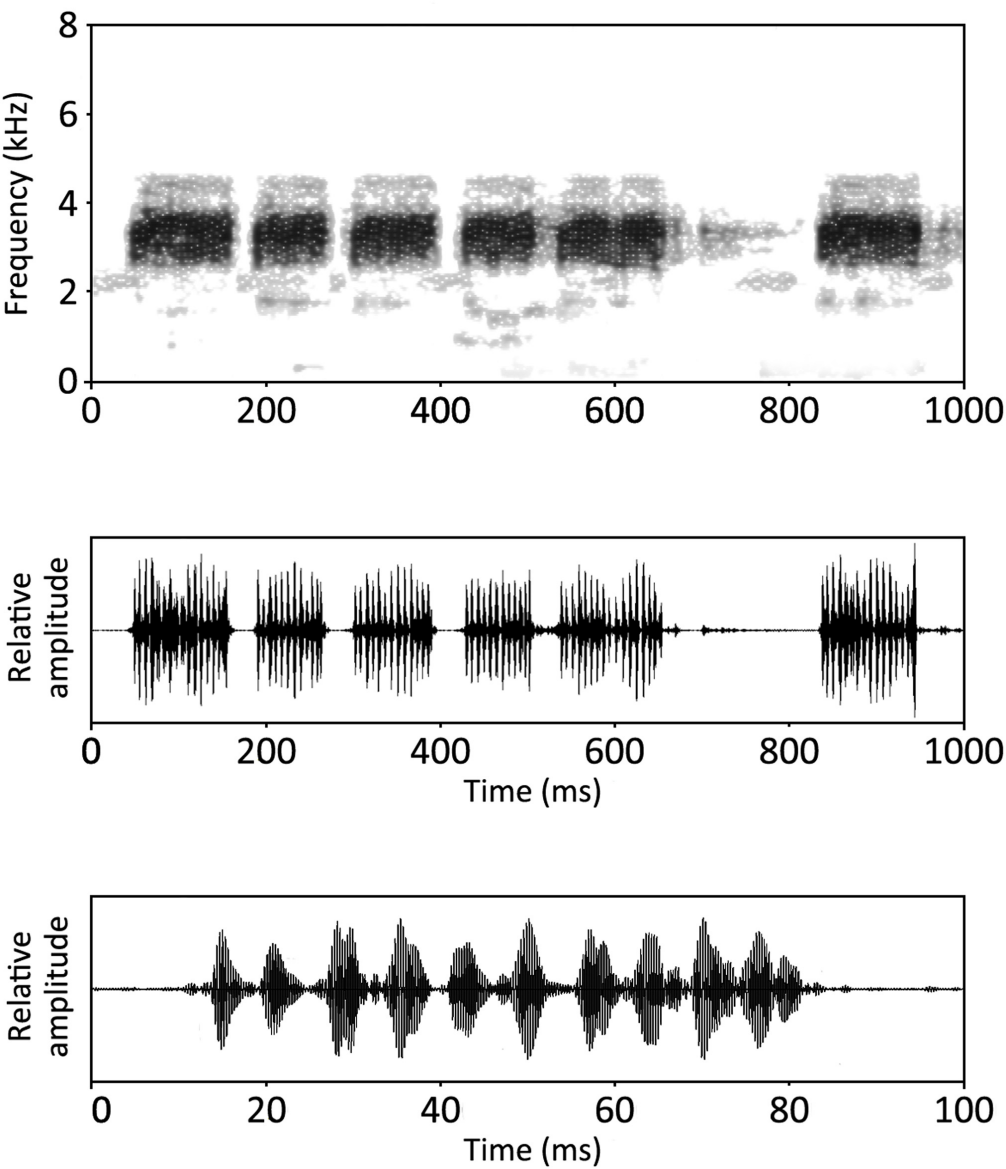
Advertisement call of *Zakerana dhaka* sp. nov. (A) Spectrogram and (B) waveform of an advertisement call. (C) A detailed image of the waveform of the second note from (B).

Advertisement calls from one male (calling on land) were recorded on June 11, 2012, in Sher-e-Bangla Nagar (Dhaka, Bangladesh) at 22:35 in air temperature of 23°C. They consisted of short chirping notes of variable duration (Fig 6). These note duration lasted between 61 to 144 ms (x = 104.2±22.5, n = 192) and were emitted singly or repeated at irregular intervals, or in short series of 2 to 16 notes (x = 4.8 ± 2.97, n = 37). Notes were arranged in series, and inter-note intervals ranged from 18 to 50 ms (x = 32.4 ± 6.7, n = 140), with intervals between two series ranging from 113 to 350 ms (x = 189.2 ± 51.5, n = 29). The number of pulses per note varied from 8 to 20 (x = 14.5 ± 3.2, n = 190) and the average pulse repetition rate was 144.5 ± 4.2 pulses/s (n = 8). The average note repetition rate was 5.7 notes/s (range: 4.6–7.7 notes/s). The call frequency ranged from 2500 to 4700 Hz with a dominant frequency lying between 2600 and 3800 Hz (Fig 6).

#### Molecular phylogenetic positioning of the new species

**Fig 7:**
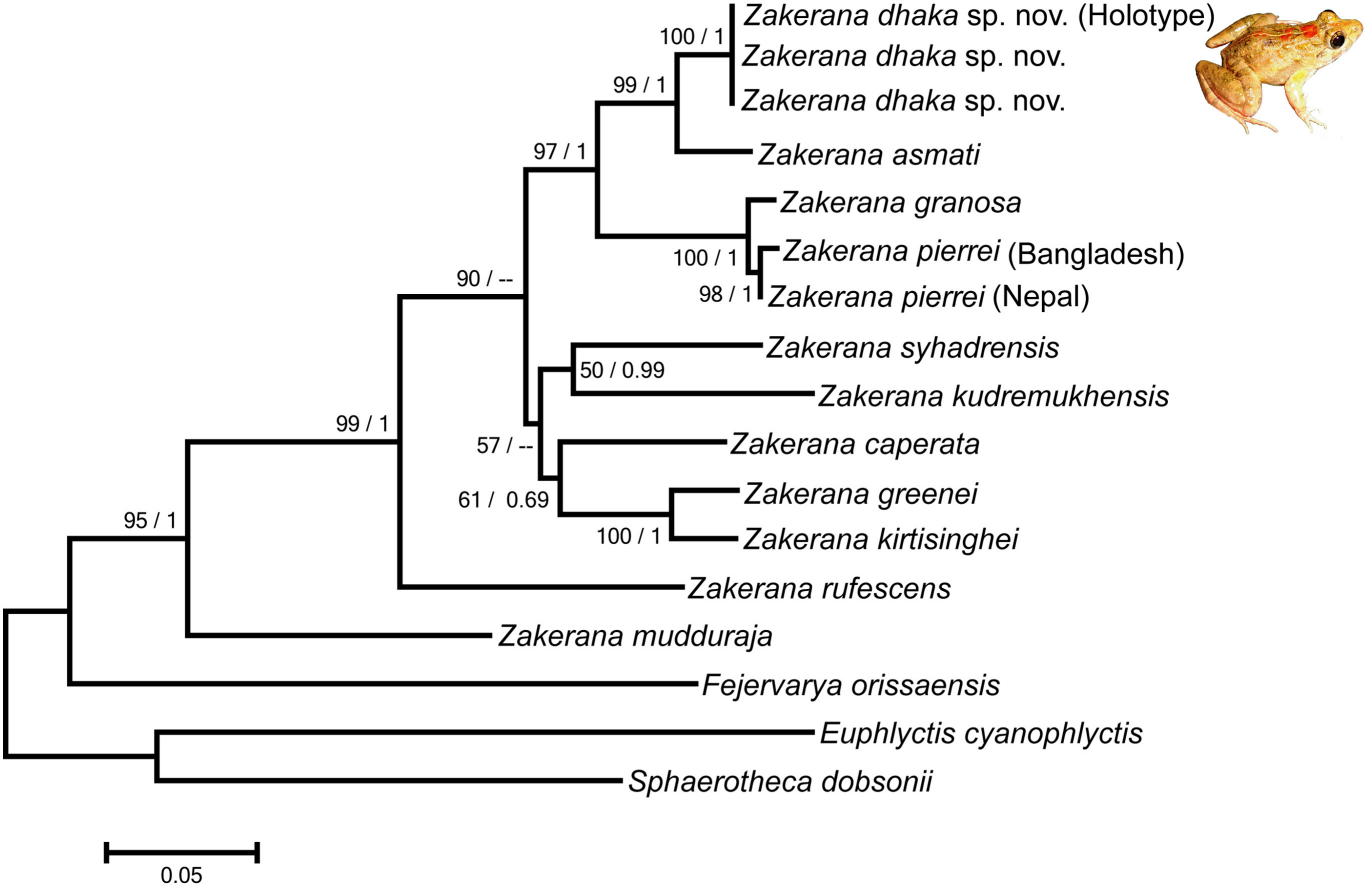
Phylogenetic relationships among species in the genus *Zakerana*. Analysis is based on 756 bp mtDNA (16S and 12S) gene sequences. Numbers on branches represent bootstrap support for Maximum-likelihood, and Bayesian posterior probability.

Maximum-likelihood and Bayesian posterior probability methods resulted in similar phylogenetic trees with a strongly supported clade for *Z*. *dhaka* (Fig 7). The new species also showed high genetic divergence from all other congeners of the genus *Zakerana*. The sequence divergences between *Z*. *dhaka* sp. nov. and other species were significant, ranging from 5–20.1% for 12S rRNA, and from 3.1–17.3% for 16S rRNA (Table 3). The intraspecific divergence was low for both genes. A sister relationship between *Z*. *dhaka* sp. nov. and *Z*. *asmati* was supported by robust bootstrap values and high posterior probabilities (Fig 7).

**Table 3:** Pairwise genetic divergences among *Zakerana* species. Lower diagonal: 16S rRNA divergence. Upper diagonal: 12S rRNA divergence.

		1	2	3	4	5	6	7	8	9	10	11	12	13
1	*Zakerana asmati*		0.0496	0.1215	0.0909	0.1041	0.1430	0.1748	0.1878	0.1036	0.1424	0.1446		
2	*Zakerana dhaka* sp. nov.	0.0311		0.1265	0.0953	0.0852	0.1352	0.1446	0.2014	0.0849	0.1435	0.1337		
3	*Zakerana greenii*	0.1029	0.1099		0.0364	0.1219	0.1250	0.1846	0.2155	0.1299	0.1313	0.1260		
4	*Zakerana kirtisinghei*	0.1064	0.1064	0.0448		0.1028	0.1347	0.1611	0.1970	0.1145	0.1233	0.1275		
5	*Zakerana pierrei*	0.0732	0.0732	0.1280	0.1280		0.1228	0.1578	0.2080	0.0177	0.1612	0.1117		
6	*Zakerana syhadrensis*	0.0892	0.0958	0.0931	0.0938	0.1129		0.1735	0.2125	0.1352	0.1206	0.1424		
7	*Zakerana rufescens*	0.1419	0.1456	0.1250	0.1353	0.1641	0.1459		0.2214	0.1492	0.1840	0.1820		
8	*Zakerana mudduraja*	0.1244	0.1456	0.1419	0.1541	0.1418	0.1527	0.1636		0.2138	0.2202	0.2315		
9	*Zakerana granosa*	0.0794	0.0794	0.1243	0.1243	0.0074	0.1165	0.1680	0.1382		0.1653	0.1081		
10	*Zakerana kudremukhensis*	0.0888	0.0986	0.1095	0.1158	0.1048	0.1022	0.1392	0.1553	0.1116		0.1473		
11	*Zakerana caperata*	0.0953	0.0986	0.0655	0.0743	0.1060	0.0709	0.1212	0.1385	0.1026	0.0922			
12	*Zakerana brevipalmata*	0.1244	0.1456	0.1419	0.1541	0.1418	0.1527	0.1636	0.0000	0.1382	0.1553	0.1385		
13	*Zakerana keralensis*	0.1422	0.1726	0.1469	0.1597	0.1468	0.1511	0.1761	0.0589	0.1401	0.1563	0.1250	0.0589	

#### Conservation

During the course of this study we observed the destruction of species habitats resulting from construction of buildings and roads. Additionally, we observed water pollution, at least partially related to garbage dumping by the Sher-e-Bangla Agricultural University and Medical Hospital. In particular, expansion of human settlements and road constructions are increasing in Dhaka, and may cause risks to the new species. Further notes on conservation status would require a more through censuses to document the actual distribution range of this species outside of Dhaka city.

## Discussion

Most species in the genera *Zakerana* and *Fejervarya* are morphologically very similar to one another and until recently, were considered to form a single genus “*Fejervarya*” ([16,17,58]. However, evidence from morphological and genetic analyses indicates the existence of two genera [16,17, 59–61]. The new species described in this study belongs to the genus *Zakerana*, and can be distinguished from all other known species in this genus on the basis of morphological and genetic characteristics. Although sequence divergence as such has been considered to be a reliable marker for amphibian species identification (e.g., [62–64]), morphological comparisons were also performed to support the genetic inference. However, consistent with earlier reports [34,65,66], the morphological divergence among species was mainly restricted to differences in body proportions. Hence, this together with the minor interest towards amphibian biology in Bangladesh, may explain why *Z*. *dhaka* has gone unrecognized as a distinct species in the middle of a mega city.

Although the genetic divergence between *Z*. *dhaka* and *Z*. *asmati* was not very high (5% for 12S rRNA, and 3.1% for 16S rRNA), it exceeded the conventional threshold value of 3% considered to be indicative of species level diverge in amphibians (e.g. [67–70]). In fact, low genetic divergence in conserved 16S rRNA gene sequence; comparable to the present study, have also been reported in many other anuran species which show distinct morphological and bioacoustic differences (e.g. [71–78]). Apart from the genetic differentiation, the fact that we found clear-cut morphometric (both quantitative and qualitative) and acoustic divergence between *Z*. *dhaka* and *Z*. *asmati* strongly suggest that they are distinct species. However, given that acoustic analyses were based on very limited material (calls of one *Z*. *dhaka* male), the strongest evidence for the species status of *Z*. *dhaka* comes from genetic and morphological data.

**Fig 8:**
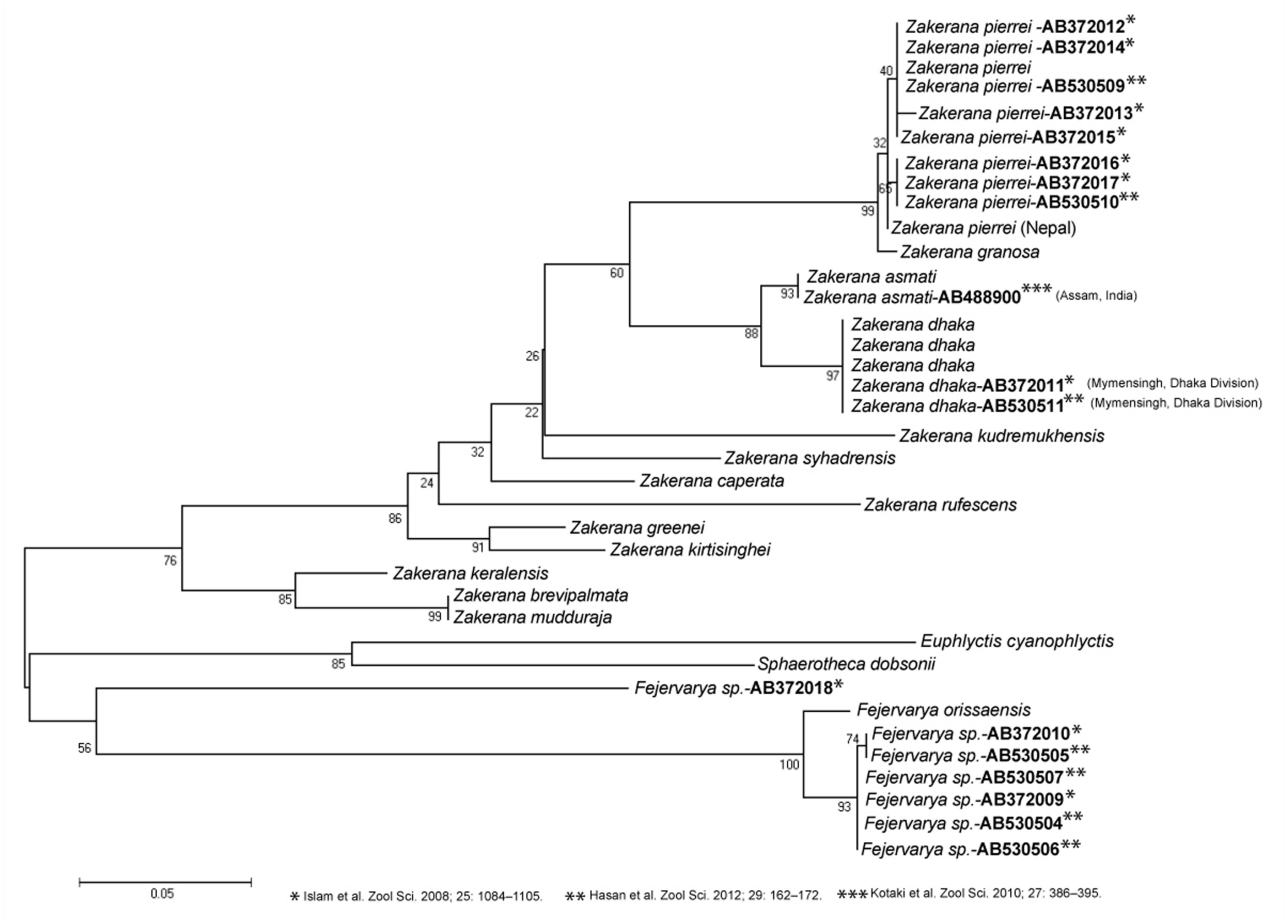
Maximum-likelihood phylogenetic tree based on variation in 16S gene fragment showing the position of *Zakerana dhaka* sp. nov. in relation to other two available haplotypes from the GenBank matched with the new species. GenBank accession numbers and locality information given after the scientific names.

Interestingly, haplotype similar to that of the new species (*Z*. *dhaka*) described here has been earlier reported by Islam *et al*. [38], and Hasan *et al*. [57] from Mymensingh of Bangladesh (Figs 1 and 8). Both Islam *et al*. [38] and Hasan *et al*. [57] identified this haplotype as “medium type *Fejervarya*”. Although Islam *et al*. [38] performed phylogenetic and morphological comparisons—and even crossing experiments among four haplotypes (*viz*. large, mangrove, medium, and small types [38])–to other species from the genera *Fejervarya* and *Zakerana*, these comparisons did not include the sister species *Z*. *asmati*. Islam *et al*. [38] ended up designating this haplotype (GenBank accession: AB372011; Fig 8) as “Bangladesh medium type”, and suggesting that only further morphological, acoustic, and genetic investigations can confirm its species status. Later on, Hasan *et al*. [57] reported that this haplotype (“*Fejervarya* sp. medium”) could be a new species, though no morphological or bioacoustics analyses were reported. Our detailed phylogenetic, morphological and bioacoustics analyses bring clarity to this debate, suggesting that the haplotype first described by Islam *et al*. [38] is in fact a new species, *Z*. *dhaka*.

Amphibians are now recognized to be the most threatened class of vertebrates [79]. Habitat loss has been identified as one of the major threats for 45% of rapidly declining amphibian species around the world [80]. Nevertheless, in the midst of these declines, the number of new amphibian species described during the past decade has been increasing (e.g. [81,82]), even from areas subject to a long history of studies in amphibian biology and systematics (e.g. [14,15]). Unfortunately, there has been a general lack of interest in amphibian taxonomy, systematics and diversity in Bangladesh during the entire 20^th^ century, possibly due to political instability, which has not nurtured interest in these types of activities. In fact, the last new amphibian species from Dhaka before the one described in this paper was reported almost 150 years ago [83]. To meet the demands of a large and growing human population, 2.1% of the country’s forests are disappearing annually [84], and more than half of the green areas and water bodies of Dhaka city have disappeared within the past 50 years [85,86]. Although the *Zakerana* species may be tolerant towards urbanization, it remains unclear how many amphibian species and populations have been lost due to these developments. If the present study is indicative of the unrecognized diversity in Bangladesh, future efforts focused on amphibian systematics and taxonomy in this country are likely to lead to the discovery of many new species, as also indicated by recent descriptions of two other new species from this country [39,40].

## Supporting Information

S1 Fig A schematic illustration of the definitions of morphological traits used in this study. See Materials and methods for explanation of trait abbreviations. Modified from Howlader et al. [2015; doi: 10.1371/journal.pone.0119825].
(TIF)

S1 Table Additional specimens examined. (PDF)

S2 Table Primers used for PCR amplifications in the present study. (PDF)

S3 Table Gene sequence identifiers (GenBank) for materials used in this study. (PDF)

S4 Table Morphological measurements of the examined specimens of *Z*. *dhaka* sp. nov. and its congeners. For trait abbreviations (columns) see Material and Methods, and for specimen identifiers (ID), see S1 Table.
(PDF)

S5 Table Loading matrix of the PCA of *Zakerana dhaka* sp. nov. and its four congeners (*Z*. *asmati*, *Z*. *pierrei*, *Z*. *nepalensis*, *Z*. *syhadrensis*, and *Z*. *teraiensis*). Factor loadings are given for the two first Eigenvectors, together with corresponding Eigenvalues and percentage of variance explained by each factor.
(PDF)
