# Supplementary figures and images for: A New Species of Frog (Anura: Dicroglossidae) Discovered from the Mega City of Dhaka
Source: PLoS One. 2016 Mar 2;11(3):e0149597. doi: 10.1371/journal.pone.0149597 (PMC4801011; doi:10.1371/journal.pone.0149597)

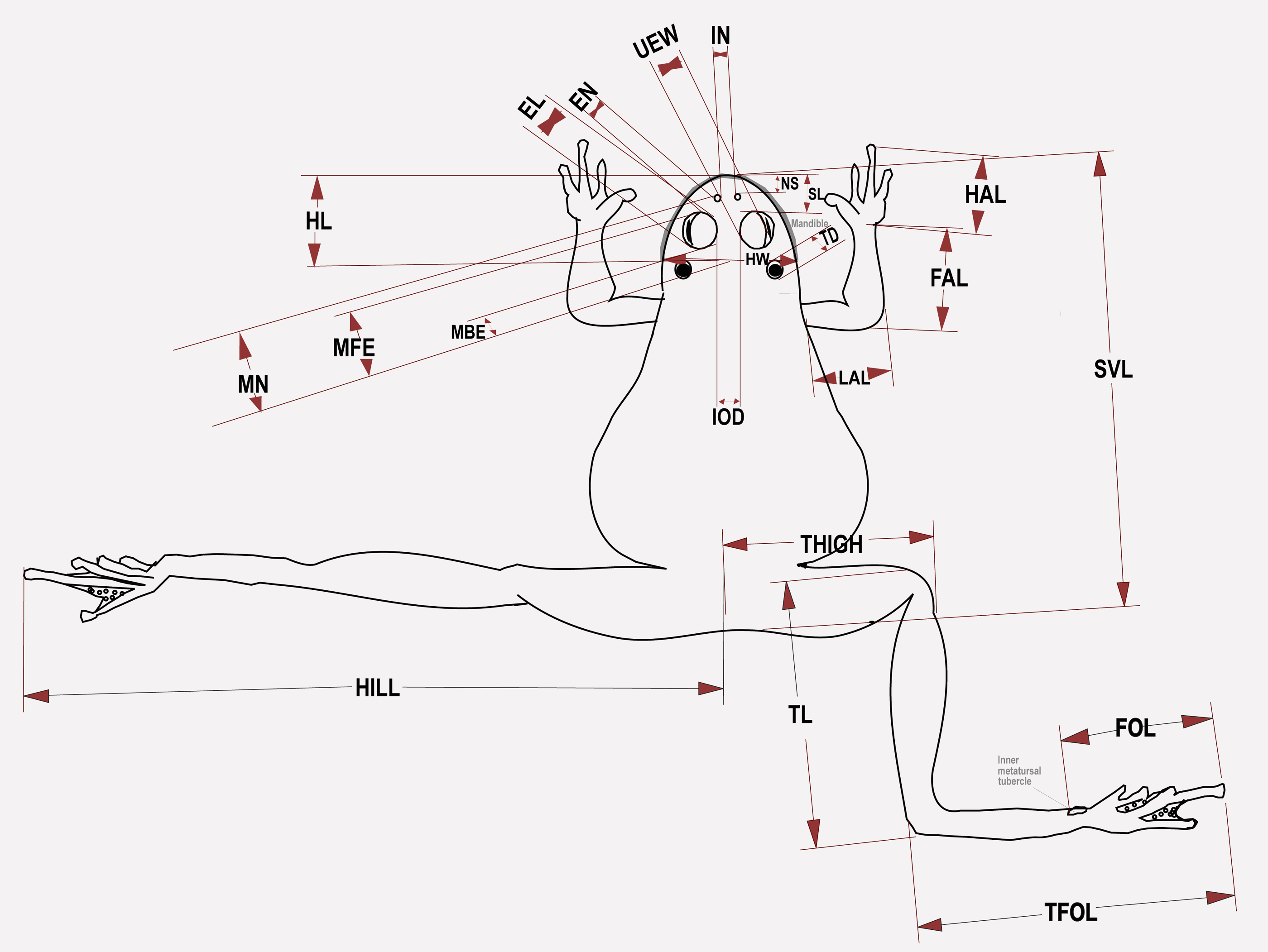

Supplement: S1 Fig — See Materials and methods for explanation of trait abbreviations. Modified from Howlader et al. [2015; doi: 10.1371/journal.pone.0119825]. (TIF) [file pone.0149597.s001.tif]
