# Supplementary material for: A New Species of Frog (Anura: Dicroglossidae) Discovered from the Mega City of Dhaka
Source: PLoS One. 2016 Mar 2;11(3):e0149597. doi: 10.1371/journal.pone.0149597 (PMC4801011; doi:10.1371/journal.pone.0149597)
Supplement: S1 Table — (PDF) [file pone.0149597.s002.pdf]

S1 Table. Additional specimens examined.

***Zakerana nepalensis* (11 specimens):** *BANGLADESH*, CHITTAGONG, Chittagong University campus: MZD/F-519, MZD/F-520, MZD/F-521, MZD/F-522, MZD/F-523, MZD/F-525, MZD/F-526, MZD/F-527, MZD/F-528, MZD/F-529, MZD/F-530.

***Zakerana pierrei* (5 specimens):** *BANGLADESH*, CHITTAGONG, Chittagong University campus: MZD/F-1201, MZD/F-1202, MZD/F-1203, MZD/F-1204, MZD/M-1205.

***Zakerana syhadrensis* (9 specimens):** *BANGLADESH*, CHITTAGONG, Chittagong University campus: MZD/F-566, MZD/F-567, MZD/F-568, MZD/F-569, MZD/F-577, MZD/F-578, MZD/F-1101, MZD/F-1102, MZD/F-1103.

***Zakerana teraiensis* (21 specimens):** *BANGLADESH*, CHITTAGONG, Chittagong University campus: MZD/F-501, MZD/F-507, MZD/F-508, MZD/F-509, MZD/F-510, MZD/F-512, MZD/F-515, MZD/F-517, MZD/F-551, MZD/F-552, MZD/F-553, MZD/F-557, MZD/F-558, MZD/F-559, MZD/F-560,MZD/F-561, MZD/F-563, MZD/F-565, MZD/F-572, MZD/F-573, MZD/F-576.

***Zakerana asmati* (6 specimens):** *BANGLADESH*, CHITTAGONG, Chittagong University campus: MZD/F-01 (Holotype), MZD/F-02, MZD/F-03, MZD/F-04, MZD/F-05, MZD/F-06.
