# Supplementary material for: A New Species of Frog (Anura: Dicroglossidae) Discovered from the Mega City of Dhaka
Source: PLoS One. 2016 Mar 2;11(3):e0149597. doi: 10.1371/journal.pone.0149597 (PMC4801011; doi:10.1371/journal.pone.0149597)
Supplement: S2 Table — (PDF) [file pone.0149597.s003.pdf]

S2 Table. Primers used in the present study for PCR amplification.

Gene	Primer	Sequence 5'- 3'	Reference
12S rRNA	FS01	5'-AACGCTAAGATGAACCCTAAAAAGTTCT-3'	Sumida *et al.* [1]
	R16M1	5'-GGGTATCTAATCCCAGTTTG-3'	Sumida *et al.* [1]
16S rRNA	F51	5'-CCCGCCTGTTTACCAAAAACAT-3'	Sumida *et al.* [1]
	R51	5'-GGTCTGAACTCAGATCACGTA-3'	Sumida *et al.* [1]

Reference:

1. Sumida M, Kondo Y, Kanamori Y, Nishioka M. Inter- and intraspecific evolutionary relationships of the rice frog *Rana limnocharis* and the allied species *R. cancrivora* inferred from crossing experiments and mitochondrial DNA sequences of the 12S and 16S rRNA genes. Molecular Phylogenetics and Evolution. 2002; 25: 293–305.
