# Supplementary material for: A New Species of Frog (Anura: Dicroglossidae) Discovered from the Mega City of Dhaka
Source: PLoS One. 2016 Mar 2;11(3):e0149597. doi: 10.1371/journal.pone.0149597 (PMC4801011; doi:10.1371/journal.pone.0149597)
Supplement: S3 Table — (PDF) [file pone.0149597.s004.pdf]

S3 Table. Gene sequence identifiers (GenBank) for materials used in study.

**Species**	**GenBank accession numbers**	
	Sequences from earlier studies	
	16S rRNA	***12S rRNA***
Zakerana greenii	AB488891	AB488868
Zakerana kirtisinghei	AB488890	AB488867
Zakerana pierrei (Nepal)	AB488888	AB488865
Zakerana syhadrensis	AB488893	AB488870
Zakerana rufescens	AB530601	AB488874
Zakerana brevipalmata	AB167946	-------------
Zakerana keralensis	GQ478322	-------------
Zakerana mudduraja	AB355833	AB355820
Zakerana granosa	AB355836	AB355823
Zakerana kudremukhensis	AB167949	AB167921
Zakerana caperata	AB355842	AB355829
Fejervarya orissaensis	AY882957	AB277289
Euphlyctis cyanophlyctis	AB167938	AB167910
Sphaerotheca dobsonii	AB277305	AB277290
	Deposited gene sequences	
	16S rRNA gene sequences	***12S rRNA gene sequences***
Zakerana pierrei (Bangladesh)	KP849816	KP849821
Zakerana asmati	KP849815	KP849820
Zakerana dhaka	KP849817, KP849818, KP849819	KP849822, KP849823, KP849824
