# Supplementary material for: A New Species of Frog (Anura: Dicroglossidae) Discovered from the Mega City of Dhaka
Source: PLoS One. 2016 Mar 2;11(3):e0149597. doi: 10.1371/journal.pone.0149597 (PMC4801011; doi:10.1371/journal.pone.0149597)
Supplement: S4 Table — For trait abbreviations (columns) see Material and Methods, and for specimen identifiers (ID), see S1 Table. (PDF) [file pone.0149597.s005.pdf]

S4 Table. Morphological measurements of the examined specimens of *Z. dhaka* and its congeners. For trait abbreviations (columns) see Material and Methods, and for specimen identifiers (ID), see S1 Table.

Museum accession	SVL	HW	HL	SL	MBE	EN	NS	EL	IN	IOD	FAL	HAL	FOL	TL
MZD/F-519	30.1	8.9	9.6	4.1	3.1	2.1	2	3.6	2.3	2.2	5.8	6.3	14.6	15
MZD/F-520	25.7	8.3	9.1	3.4	2	1.8	1.6	3.3	2.1	1.5	3.9	5.7	13.4	13
MZD/F-521	31.2	9.6	10.3	3.5	3.3	2.4	1.5	3.2	2.6	2.5	6.4	6.4	15.7	16.4
MZD/F-522	26.3	8.2	9	3.5	2.3	1.9	1.6	3.4	2.1	1.7	4.3	6	13.2	13.1
MZD/F-523	22.5	7.3	8.3	3	2.2	1.7	1.3	2.5	2	1.7	4	5.3	11	12
MZD/F-525	25.6	8.3	8.6	3.8	2.5	2.3	1.5	3.3	2.3	2.2	4.5	5.4	14.2	14.5
MZD/F-526	26.6	7.9	8.7	3.5	2.5	2.1	1.4	3.4	2.1	2.1	4.8	5.2	13.8	14
MZD/F-527	24.1	8	8.5	3.7	2.5	2.3	1.4	3	2.3	2.3	5.2	5.2	12.8	13.6
MZD/F-528	26.4	8.3	8.7	3.9	2.8	2.2	1.7	3.2	2.4	2.2	4.8	5	13.2	13
MZD/F-529	22.7	7.7	8.2	4	2.6	2.3	1.7	3.3	2.4	2.2	5	5.7	12.8	12.4
MZD/F-530	21.4	7.3	7.5	3.7	1.8	2.1	1.6	2.4	2.2	2.2	4.3	5.4	11.8	11.7
MZD/F-1201	29.4	9	9.7	3.9	2.9	2	1.9	3	2.3	2	5.2	6.2	15.3	15.4
MZD/F-1202	23.1	7.2	7.7	3.2	2.5	1.7	1.5	3	2	1.6	4.5	5.6	13	11.3
MZD/F-1203	29.4	9.1	9.4	4.4	2.8	2.4	2	3.5	2.3	1.9	4.9	5.8	15.4	15
MZD/F-1204	25.6	7.5	7.7	3.4	2.4	1.8	1.6	2.6	1.9	1.8	4.5	5.3	13	13.1
MZD/F-1205	27.8	9	9.1	3.8	2.7	2.1	1.7	3.1	2	1.9	5.1	5.9	14.3	14.5
MZD/F-566	28.8	9.2	9.6	4.1	3.2	2.4	1.7	3.2	3	2	5.5	5.5	16	15
MZD/F-567	32.8	9.7	10.5	5.4	3.3	3.1	2.3	3.9	3	2	6.5	6.8	16.5	17
MZD/F-568	28.8	9.1	9.4	4.6	3.3	2.2	2.4	3.2	3.3	2	6	6	16.5	15.7
MZD/F-569	27.1	8.1	8.8	4.5	2.8	2.3	2.2	3.2	2.6	1.7	5	5.2	12.1	14.3
MZD/F-577	28.1	9.2	9.1	4.3	2.6	2.3	2	3.4	2.8	2.2	5.6	5.6	14.6	13.5
MZD/F-578	28.5	9	9	4.4	2.4	2.3	2.1	3.6	2.5	2.4	5.8	5.7	15.6	15
MZD/F-1101	37.2	11.7	12	5.2	4.6	2.9	2.3	3.8	3.5	2.4	7	7.4	21.2	20.6
MZD/F-1102	30	9.3	9.6	4	3	2.2	1.8	3.7	2.5	2	5.7	5.7	15.4	15.4
MZD/F-1103	33.4	11.3	11.6	4.5	3.4	2.3	2.2	3.4	3.3	1.8	7.2	7.2	17.6	17.5
MZD/F-501	40.7	14.5	14.6	6.5	4.9	3.1	3.4	4.6	2.6	2	8.5	9	20.8	20.8
MZD/F-507	44.8	14	14	6.4	5.1	3.2	3.2	4.6	3.5	2.7	7.8	7.8	19.5	21.5
MZD/F-508	45.8	14.4	14.4	7.1	5.4	3.4	3.7	5	3.8	2.1	8.7	8.6	21.4	20
MZD/F-509	50.1	16.4	16.4	6.5	6.2	3.7	2.8	5.2	3.2	2.5	10	10.1	19.6	24.6
MZD/F-510	41.5	13.1	13.2	6.2	4.7	3.2	3	4.6	3	2.1	7	6.8	18.7	19.6
MZD/F-512	37.8	12.4	12.8	6.3	4.3	3.2	3.1	4.5	3.2	2.3	7.5	7.5	19.3	19.3
MZD/F-515	47.8	15.6	15.6	6.7	5.6	3.4	3.3	5.5	3.7	2.3	9.1	9.1	22.5	22.5
MZD/F-517	42.5	13.8	13.8	6.8	3.7	3.5	3.3	5.1	3.1	2.9	9	9	21.4	22
MZD/F-551	43.8	13.9	13.9	6.9	4.8	3.6	3.3	4.7	3.5	2.8	8.3	8.3	20	22.1
MZD/F-552	52.3	16.8	17.3	7.4	6.4	3.7	3.7	5.3	3.5	2.2	10.5	10.6	26	27.3
MZD/F-553	48	14.4	14.8	6.7	4.8	3.5	3.2	4.4	3.8	2.1	9.3	8.6	20.7	22.7
MZD/F-557	44	13.9	13.9	6.4	5.1	3.2	3.2	4.1	3.5	2.2	8.1	8.1	19.5	20.3
MZD/F-558	49.1	15.6	16	6.9	4.6	3.4	3.5	5.4	3.6	2	9.5	9.6	23.9	24
MZD/F-559	46.8	16	15.2	6.8	4.6	3.5	3.3	5.3	3.4	2.5	9.5	9.8	24	25.7
MZD/F-560	41.5	13.7	13.2	6.4	4.5	3.3	3.1	4.6	3.1	2.4	8	8	19.8	20.4
MZD/F-561	47.6	15.7	16.2	6.8	5.4	3.7	3.1	5.4	3.5	2.5	9.6	9.6	24.1	25
MZD/F-563	48.1	15.4	15.4	6.7	4.8	3.4	3.3	5	3.2	2.6	8.7	7.2	21.7	23.2
MZD/F-565	42.1	13.8	13.8	6.4	4.2	3.4	3	4.7	3.1	2.8	7.6	8.3	19.8	19.6
MZD/F-572	35.7	12.5	12.3	5.1	2.8	2.5	2.6	4	3.4	1.8	6.8	7.3	17	17.3
MZD/F-573	51.4	17	16.8	6.7	6.9	3.5	3.2	5.4	3.2	2.3	10.3	8.1	25.3	25.4
MZD/F-576	36.2	11.5	12.4	5.4	4.2	2.6	2.8	4.1	3	1.8	7.3	7.3	16.3	18.3
MZD/F-01	30	9.6	10.3	4.1	1.9	2.7	1.5	4.3	2.3	2	5.1	7.2	15.7	15.1
MZD/F-02	33.4	10.5	11.4	4.6	2.2	2.8	1.7	4.7	2.4	2.1	5.8	7.6	16.7	16.1
MZD/F-03	32.2	10.9	11.8	4.6	2.1	3.0	1.7	5.0	2.5	2.2	5.1	7.6	16.7	15.9
MZD/F-04	30.5	11.0	11.5	4.3	2.0	2.6	1.5	4.6	2.2	1.9	5.0	7.3	16.0	15.5
MZD/F-05	31.0	10.9	11.9	4.5	2.3	2.9	1.6	5.1	2.4	2.2	4.7	7.4	16.0	15.4
MZD/F-06	29.1	9.3	10.0	4.0	1.8	2.6	1.5	4.2	2.2	1.9	5.3	7.5	15.8	15.2
MZH-3371	30.5	10.7	11.1	4.4	2.8	2.3	1.8	4.6	2.8	1.8	7.6	13.9	16.6	19.7
MZH-3373	30.2	10.3	10.6	4.2	2.4	2.1	1.8	4.2	2.8	1.8	7.6	13.7	16.3	19.6
MZD-1031	30.0	10.2	10.8	4.4	2.5	2.2	1.7	4.6	2.8	1.8	7.6	13.6	16.1	19.7
MZD -1032	29.2	10.2	9.9	4.0	2.2	1.9	1.6	4.1	2.6	1.6	7.4	13.5	16.5	19.6
MZD -1033	28.2	9.9	9.6	3.9	2.2	2.2	1.7	3.8	2.8	1.8	7.0	13.4	15.9	19.6
MZD -1034	27.8	10.0	9.7	4.2	2.1	2.1	1.6	4.1	2.6	1.6	6.8	13.1	16.4	19.5
MZD -1035	28.2	10.3	9.9	3.9	2.3	2.0	1.7	3.9	2.6	1.7	7.1	13.6	15.9	19.6
MZD -1036	31.6	12.0	11.7	4.7	2.6	2.1	1.8	4.7	2.8	1.7	7.6	13.1	17.0	19.8
MZD -1037	27.8	9.7	9.5	3.8	2.4	2.1	1.7	4.1	2.8	1.7	6.9	13.3	16.2	19.5
MZH-3372	37.5	11.9	12.6	4.8	2.8	2.6	2.1	5.2	3.6	2.4	9.2	16.9	19.9	24.3
MZD -1038	37.9	14.2	13.7	5.5	3.0	2.7	2.1	5.6	3.5	2.2	9.0	16.9	19.7	24.4
MZD -1039	33.5	12.1	11.7	4.6	2.9	2.5	2.0	4.7	3.4	2.1	9.1	16.5	20.0	24.1
