# Supplementary material for: A New Species of Frog (Anura: Dicroglossidae) Discovered from the Mega City of Dhaka
Source: PLoS One. 2016 Mar 2;11(3):e0149597. doi: 10.1371/journal.pone.0149597 (PMC4801011; doi:10.1371/journal.pone.0149597)
Supplement: S5 Table — Factor loadings are given for the two first Eigenvectors, together with corresponding Eigenvalues and percentage of variance explained by each factor. (PDF) [file pone.0149597.s006.pdf]

S5 Table. Loading matrix of the PCA analysis of *Zakerana dhaka* sp. nov and its four congeners (*Z. asmati*, *Z. pierrei*, *Z. nepalensis*, *Z. syhadrensis*, and *Z. teraiensis*). Factor loading are given for the two first Eigenvectors, together with corresponding Eigenvalues and percentage of variance explained by each factor.

Trait	Principal component 1	Principal component 2
**SVL**	0.979	-0.116
**HW**	0.982	0.033
**HL**	0.976	-0.012
**SL**	0.960	-0.191
**MBE**	0.874	-0.306
**EN**	0.912	-0.285
**NS**	0.902	-0.274
**EL**	0.848	0.292
**IN**	0.863	0.111
**IOD**	0.584	-0.468
**FAL**	0.930	0.283
**HAL**	0.369	0.902
**FOL**	0.964	0.067
**TL**	0.917	0.349
**Eigenvalue**	10.78	1.63
**% variance explained**	77.0	11.7
